# Lipophilicity as a Central Component of Drug-Like Properties of Chalchones and Flavonoid Derivatives

**DOI:** 10.3390/molecules24081505

**Published:** 2019-04-17

**Authors:** Teodora Constantinescu, Claudiu Nicolae Lungu, Ildiko Lung

**Affiliations:** 1 Department of Chemistry, Faculty of Pharmacy, Iuliu Hatieganu University, 400012 Cluj-Napoca, Romania; teo_farma@yahoo.com; 2 Department of Chemistry, Faculty of Chemistry and Chemical Engineering, Babes-Bolyai University, 400028 Cluj-Napoca, Romania; 3 National Institute for Research & Development of Isotopic and Molecular Technologies 67-103 Donath street, 400293 Cluj-Napoca, Romania; ildiko.lung@itim-cj.ro

**Keywords:** lipophilicity, retention factor, chalcones, QSAR, chromatography, drug design

## Abstract

Lipophilcity is an important physico-chemical parameter that influences membrane transport and binding ability to action. Migration distance following complete elution of compounds was used to calculate different lipophilicity-related parameters. The aim of this study is to show that lipophilicity is a central component of thiazole chalcones and flavonoid derivatives regarding their drug-like properties. Experimental and computational methods were used. This study considers 44 previously synthesized compounds (thiazole chalcones, flavanones, flavones, 3-hydroxyflavones, and their acetylated derivatives). The concerned compounds have shown antitumoral hallmarks and antibacterial activity in vitro. The experimental method used to determine compounds’ lipophilicity was the reverse-phase thin layer chromatography (RP-TLC). Lipophilicity related parameters—isocratic retention factor (R_M_), relative lipophily (R_M_^0^), slope (*b*), chromatographic hydrophobic index (φ_0_), scores of principal components (PC1/R_M_)—were determined based on reverse-phase chromatography results.

## 1. Introduction

Lipophilicity is an important feature of molecules in pharmaceutical, biochemical, and medical chemistry fields. Lipophilicity applications include drug design, route of administration and chromatographic separation. The hypothesis of pH-partition asserts that the absorption of ionizable drugs takes place where the local pH provides the maximum concentration of the non-ionized form relative to the ionized form concentration. In addition, lipophilicity is a physico-chemical parameter that affects the affinity of a molecule for binding sites and passive transport through biological membranes [1,2,3,4]. Affinity of molecules for a medium is often determined as a partition coefficient between water and an immiscible solvent, expressed as a decimal logarithm of the partition coefficient (log P) in two non-miscible solvents (e.g., water–isopropanol, water–*n*-octanol) [5,6,7]. Partition coefficient is often used in structure–activity relationships (SAR) and quantitative structure–activity relationships (QSAR) [8,9]. Some studies have described significant relationships between the determination of lipophilic parameters and the chemical structure, bioactivity, and the pharmacokinetic properties of the biologically active compounds [10]. Lipophilicity of a drug molecule is of particular importance due to its impact on the metabolism, pharmacokinetics, pharmacodynamics, and molecular toxicity of molecules. It correlates with the absorption, distribution, metabolism, excretion, and molecule toxicology (ADMET) processes [11,12,13,14,15,16].

Generally, there are two classes of methods developed for log P determination: Computational and experimental. There are many computational variants for predicting this property, using simple methods based on a small number of descriptors up to sophisticated neural network algorithms and involving thousands of correction factors. These methods can be greatly improved by local corrections; it is essential to compare the results with those provided by experimental methods [17,18].

Although RP-HPLC represents a very good method because of its good accuracy, low sample consumption, on-line detection, and its ability to perform measurements even in a presence of a mixture, the reverse-phase thin layer chromatography (RP-TLC) method has numerous advantages such as simplicity of the equipment, extremely low mobile phase usage, high transfer rate, and low cost of analysis. The RP-TLC method can also be used for compounds with increased lipophilicity. To describe the solute retention constants, the R_M_ parameter shows a very good linear correlation with log P for neutral and ionic compounds. Another parameter of hydrophobicity is R_M_^0^, sometimes preferred to the R_M_ parameter. R_M_^0^ is the 0% *v/v* extrapolated value of the organic modifier for estimating the solute partition between water and the non-polar stationary phase as measured in lipophilic solute. Another parameter, C_0_, expresses the concentration of an organic change in the mobile phase for which the distribution of the solute between the two phases is equal. One of the essential elements of the chromatographic system, the mobile phase, has a significant role in chromatographic behavior of different solvents [2,19,20].

The term chalcone is used to name compounds characterized by the presence of 1,3-diphenyl-2-propen-1-one subunit [21]. Chalcones and their derivatives exhibit many activities (anticancer, anti-inflammatory, antioxidant, cytotoxic, antibacterial, analgesic, antipyretic, antihepatotoxic, antimalarial, and anti-allergic) [22,23,24]. Chalcones play the role of pharmacophore in many natural products such as cumarin, flavokawain, milepachin, and xanthohumol [25]. Flavanones, 2-aryl chroman-4-one, have numerous biological properties (anticancer, antimicrobial, anti-inflammatory, antiviral, etc.) [26]. Flavones or 2-phenylchromones are present in many natural products and represent a significant group of oxygen heterocycles, present in plants as secondary metabolites. The synthesis of flavones has a particular interest due to the biological activities of these compounds—anti-inflammatory, anti-estrogenic, antioxidant, anti-cancer, antiretroviral, antihypertensive, antimicrobial, anti-diabetic, anti-allergic, and chemopreventive properties have promoted numerous ways in flavones synthesis [9,27].

The thiazole nucleus is an important component for a large number of therapeutic agents with anticancer, anticonvulsant, antifungal, and antibacterial properties. The heterocycle presents applications for the synthesis of new biologically active compounds with cardiotonic, fungicidal, and antiretroviral properties. Also, thiazole may be a subunit used in the synthesis of novel molecules used in the treatment of neurodegenerative diseases (Parkinson’s, Alzheimer’s). This class of heterocyclic compounds is present in numerous biologically active molecules, such as sulfatiazole (antimicrobial), ritonavir (antiretroviral), bleomycin (anticancer), and meloxicam (anti-inflammatory) [6,28].

Since flavonoids and thiazole compounds exhibit numerous biological activities, and lipophilicity is an essential parameter for pharmacokinetics, pharmacodynamics, and molecular toxicology, the aim of the current study was to determinate the lipophilicity of 44 compounds (thiazole chalcones, flavanones, flavones, 3-hydroxyflavones, and their acetylated derivatives) previously synthesized by us using experimental and computational methods in order to evaluate this central component of drug-like properties. Keeping in mind the antitumoral activity of the synthesized compounds, lipofilicity is an important parameter to indicate whether these compounds may constitute a starting point for the development of novel antitumoral agents.

## 2. Results and Discussion

### 2.1. Chromatographic Evaluation

The structure of 20 chalcones (**1a**–**t**) was substituted with hydroxy or methoxy groups in *ortho* and/or *para* positions of acetophenone and with electron donor groups (methoxy, metyl), unsubstituted or substituted with electron acceptor groups (chlorine) in the *para*-position of the phenylthiazole. The basic structure is that of 1,3-difenyl-2-propen-1-one, then the retention differences are attributed to functional groups and their positions at level of the two subunits. In the case of hydroxychalcones, retention capacity is influenced by the nature of substituents at *para*-position of phenylthiazole. For *para* hydroxychalcones, the highest retention capacity is represented by chlorine-substituted chalcone (**1c**) in *para* position of phenylthiazole, followed by methyl-substituted chalcone (**1b**) and unsubstituted phenyl, respectively (**1a**). The lowest retention capacity is represented by chalcone substituted with a methoxy group (**1d**). Compared to the chalcone substituted in *ortho* position of acetophenone, chalcones with a hydroxy group in *para* position have a lower retention capacity and a higher hydrophilic character. This can be explained by the ability of *ortho* hydroxylchalcones to form hydrogen-bonding bonds with the carbonyl group on the propene-2-one moiety. In the series of *ortho* hydroxychalcones, the highest retention capacity is shown by the chlorine-substituted compound in *para* position of phenylthiazole (**1g**). As with *para* hydroxychalcones, retention capacity of *ortho* hydroxychalcones varies in the order **1g** > **1f** > **1e** > **1h**. Substitution with a methoxy group increases hydrophilia of the molecule.

For *para* methoxychalcones, the highest retention capacity was shown by *para* chlorine-substituted chalcone (**1k**) of phenylthiazole; the order of increasing in retention capacity is **1k** > **1j** > **1i** > **1l**. The most hydrophilic *para*methoxychalcone is the one substituted with a methoxy group on phenylthiazole (**1l**). For *ortho*, *para* dimethoxychalcones (**1m**–**p**), and *ortho* methoxychalcones, the order of decreasing retention capacity is similar to that of *para* methoxychalcones. Among methoxychalcones, the highest retention capacity was in *para* methoxychalcones, followed by *ortho* methoxychalcones, respectively. Inclusion of a methoxy group on acetophenone results in decreases in retention capacity. Replacement of hydroxy group in *para* position of acetophenone with methoxy determines the increase of retention capacity due to decrease in its polarity. When replacing hydroxyl group with methoxyl in *ortho* position of chalcones, the result is a decrease in retention capacity. This is explained by the bonds formed between *ortho* substituent of acetophenone and the carbonyl group. Among previously synthesized chalcones, the lowest retention capacity was observed in *para* hydroxychalcones. Compound **1d** is the most hydrophobic of the 20 thiazole chalcones.

In the synthesized thiazole flavanones (**2a**–**d**), lipophilicity is influenced by the nature of the substituent in *para* position of phenylthiazole. The basic subunit of flavanones, 2-aryl-chroman-4-one, changes the retention capacity compared to *ortho*hidroxychalcones from which they are synthesized; the order of increase in retention capacity is **2d** < **2a** < **2b** < **2c**. The highest retention capacity is represented by chlorine-substituted flavanone in *para* position of phenylthiazole (**2c**); this represents the most lipophilic flavanone. The most hydrophilic flavanone is the compound with a methoxy group in *para* position of phenylthiazole (**2d**).

Flavones and 3-hydroxyflavones (**3a**–**h**) have the common structure of 2-aryl-chromen-4-one. Lipophilicity of molecules depends on the nature of substituents in *para* position of phenylthiazole and on the presence or absence of hydroxy group in position 3 of chromene. For thiazole flavones, the retention capacity of compounds increased in the order **3c** > **3b** ≥ **3d** > **3a**. The highest retention capacity is shown by chlorine-substituted flavone in *para* position of phenylthiazole (**3c**). Unsubstituted flavones on phenylthiazole are the most hydrophilic (**3a**). In the case of 3-hydroxyflavones, the highest retention capacity was observed at the compound substituted by chlorine on phenylthiazole (**3g**). The most hydrophilic compound is the unsubstituted on phenylthiazole (**3e**). Compared to flavones, 3-hydroxyflavones have a lower retention capacity due to hydrophilicity, determined by the hydroxyl group.

In the case of acetylated derivatives (**4a**–**l**), obtained by acetylation of hydroxyl groups, a decrease in polarity of compounds and a decrease in retention capacity in reverse phase determinations was observed. For this reason, experimental determination of lipophilicity was performed at other concentrations of isopropanol, by water mixture.

For acetylated derivatives, the lipophilicity of the compounds is influenced by the basic structure of the molecules (1-3-difenyl-2-propen-1-one or 3-hydroxyflavones) and the nature of *para* substituents of the phenylthiazole. In case of chalcones derivatives, lipophilicity is also influenced by the position of acetate group on acetophenone. Retention capacity varied: **4c** ≥ **4b** > **4g** > **4k** > **4f** > **4a** > **4d** ≥ **4j** > **4e** > **4h** > **4i** > **4l**. The higher retention capacity was shown by the acetylated derivatives of chlorine-substituted *para* hydroxychalcone in *para* position of phenylthiazole (**4c**), and the lower was the methoxy substituted 3-hydroxyflavone derivative in *para* position of phenylthiazole (**4h**).

### 2.2. Computational Evaluation

**Figure 1:**
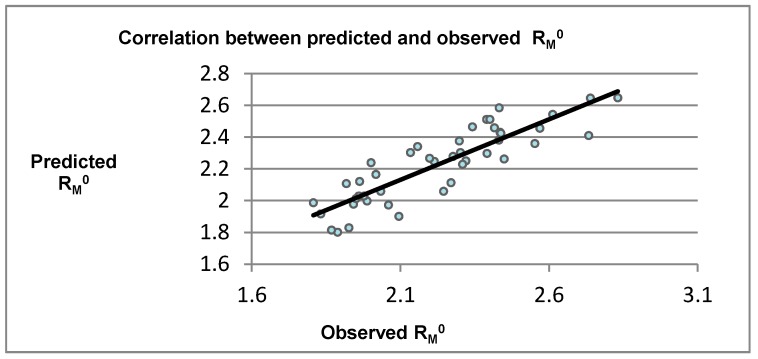
Plot showing correlation between observed and predicted R_M_^0^, using multiple linear regression (MLR).

In order to evaluate correlation between lipophilicity and compounds structure, a multiple linear regression (MLR) model was developed (Figure 1) [29]. R_M_^0^ was chosen as dependent variable, while for independent variables, a variety of molecular descriptors were used accordingly with Topoliss–Costello rule. Data were split randomly, into a training set and a dataset equally. The model was internally and externally validated (see Supplemental Materials). S, R^2^, and φ^0^ have also been considered, but correlations obtained were not as high as for R_M_^0^. The obtained model had a Pearson correlation r = 0.873, Pearson correlation squared r^2^ = 0.7619**,** Spearman rank correlation ρ = 0.874, cross validated square q = 0.761, mean squared deviation (MSD) = 0.0162, and y = 0.530798 + 0.761964x. Interaction model using neural network regression (NNR), (random seed 3449287299, max training epochs 1000, learning rate 0.30, output layer learning rate 0.30, number of neurons first hidden layer = 3, using N-fold cross validation N = 10, percentage split 66), retrieved a Pearson correlation of r = 0.971.

**Figure 2:**
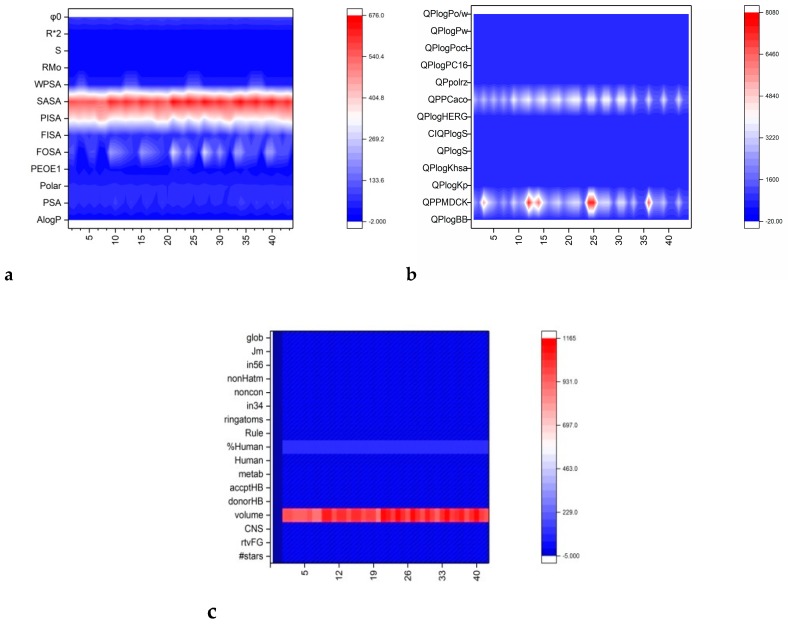
Compounds’ (#44) lipophilicity and drug-like properties represented as heatmaps; (**a**) RMo values for each compound represented together with some computed lipophilicity-related descriptors. (**b**) QPlog-related properties for each compound. (**c**) Drug-like properties for each compound.

Based on reported studies, and the difference in r^2^ obtained by MLR and NNR (that suggest the presence of another parameter that correlates with R_M_), principal component analysis (PCA) was performed. Principal components (PC) for R_M_ were computed [30]. Using PC values, a new parameter, which presumably contained relevant information was created by dividing each PC to R_M_ [31]. This newly obtained parameter was used in the clustering technique in order to identify common characteristics in the 44 studied compounds regarding lipophilicity. The lipophilicity space was computed using the clustering variable as the z coordinate, and the x and y variables were the parameters with the highest variability in respect to lipophilicity (see Figure 2).

Lipophilicity related parameters—isocratic retention factor (R_M_), relative lipophily (R_M_^0^), slope (*b*), chromatographic hydrophobic index (φ_0_), together with some computed descriptors—are represented in Figure 2a.

A component of total solvent accessible surface area (SASA) was computed for N, O, H on heteroatoms (FISA), hydrophobic component of the SASA (FOSA), total positive van der Waals surface area (PEOE1), polarizability (polar), polar surface area (PSA), and partition coefficient (AlogP). SASA and FOSA showed considerable variability along the set of compounds. All these parameters are in the range of druggability (Figure 2a). QPlog-related parameters, like lipophilicity, showed relatively constant values for all compounds. Exceptions were noted at the predicted apparent Caco-2 cell permeability, in nm/s (QPPCaco), and predicted apparent Mandin Darby Canine Kidney MDCK cell permeability, in nm/s (QPPMDCK). Overall compounds showed excellent membrane permeability-predicted properties (Figure 2b).

Drug-like properties, both experimental and computational, are excellent. There were no violations of drug-like criteria. Drug-like properties computed proved to be constant for all compounds with one exception: The molecular volume of each compound apparently had no effect on druggability. Also, % human oral absorption, even if it had a constant value along the compound series, had a different dimensionality when compared with the other parameters (Figure 2c).

**Figure 3:**
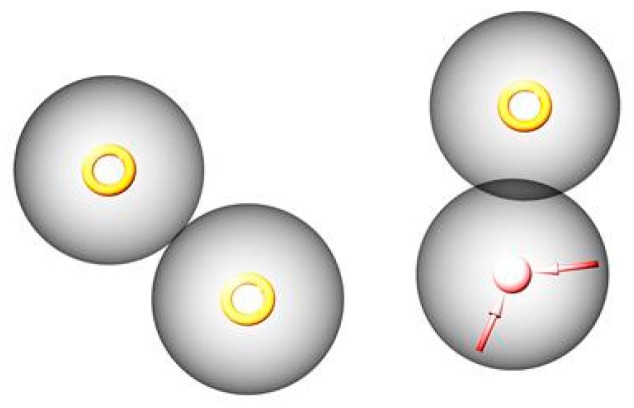
Pharmacophore model. Hydrogen acceptor—pink arrow. Aromatic groups—orange rings.

A pharmacophore model was computed by setting R_M_^0^ as the dependent variable in order to explain 90% of the observed R_M_^0^ values. The model was constructed using ARRR hypothesis (A-H acceptor; R-aromatic ring). Model cartesian coordinates are: x −4.12, y −1.95, z 0.00R5x −0.03, y −1.98, z 0.00; A2x 5.29, y 0.75, z 0.00R8x 2.75, y 3.49, z 0.00. The pharmacophore model is showed in Figure 3.

**Figure 4:**
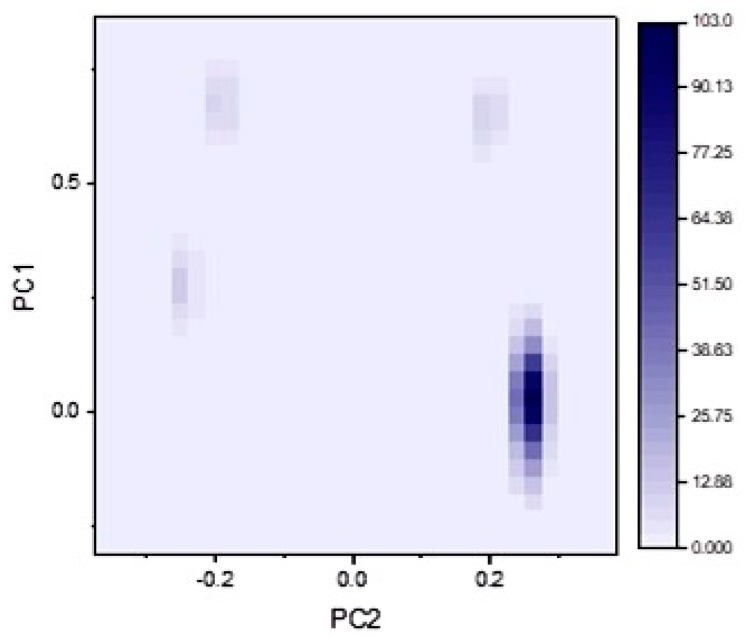
Heat map showing density clustering of all retention factors for thiazole chalcone and flavone derivatives. One main cluster is observed consecutively with three minor ones.

Clustering based on R_M_^0^ principal component analysis was performed (Figure 4). PC1 and PC2 was computed. Hierarchical clustering analysis showed four clusters corresponding to the discussed retention factors. Clusters obtained are similar to the peaks obtained by representing R_M_^0^ for all compounds as a scatter plot (see Supplementary Materials). Acetylated compounds seem to form a distinct strong group of clusters with higher densities.

## 3. Materials and Methods

Thiazole chalcones, flavanones, flavones, 3-hydroxyflavones, and their acetylated derivatives have been previously synthesized by us. Thiazole chalcones were obtained by Claisen-Schmidt condensation of thiazole aldehydes with substituted acetophenones in *ortho* and/or *para* positions with hydroxy or methoxy groups [32]. By the cyclization of *ortho* hydroxychalcones with concentrated sulphuric acid [33], sodium acetate [34], or glacial acetic acid [35], the corresponding thiazole flavanones were synthesized**.** Flavones were obtained by oxidative cyclization of *ortho* hydroxyl/methoxychalcones with iodine in dimethylsulfoxide [36]. The 3-hydroxyflavones were synthesized by using urea-hydrogen peroxide complex or hydrogen peroxide in the presence of sodium hydroxide [37]. The acetylated derivatives of hydroxyl substituted compounds were obtained by treatment with acetic anhydride in the presence of pyridine [38]. The structure of synthesized compounds is presented in Supplementary Materials.

### 3.1. Experimental Evaluation

Reversed-phase thin layer chromatography (RP-TLC) was carried out using aluminum plates silica gel coated with fluorescent indicator RP-18F254s. The plates were purchased from Merck Millipore. Spots were visualized in UV light at 254 and 365 nm. Analytical grade isopropanol used in the experiment was purchased from Merk. The retention factor (R*_f_*) values obtained by RP-TLC were used for R_M_ parameters (isocratic retention factor) and were calculated with the Bate-Smith and Westall equation [11]:

R_M_ = log[(1/R*_f_*) − 1]. (1)

Based on the linear relationship between R_M_ values and the concentration of the organic solvent in the mobile phase (Sosczewinski–Wachtmeister equation), three lipophilicity parameters were calculated: (a) R_M_^0^ (which corresponds to 0% methanol in the mobile phase); (b) which represents the slope, and (c) which represents the volume fraction of methanol in the mobile phase [11]: R_M_ = R_M_^0^ + *b*C

The chromatography hydrophobic index, φ_0_, was calculated using the following equation [19]:

φ_0_ = R_M_^0^/*b*. (2)

RP-TLC experiments were performed for 20 thiazole chalcones, 4 flavanones, 4 flavones, 4 3-hydroxyflavones, and 12 acetylated derivatives. Compounds were divided in 3 groups: (1) thiazole chalcones with hydroxyl or methoxy groups (20 compond, **1a**–**t**), (2) thiazole flavanones, flavones, and 3-hydroxyflavones (12 compounds, **2a**–**d**, **3a**–**h**), and (3) acetylated derivatives (12 compounds, **4a**–**l**). TLC plates were prepared using a stationary phase and a mixture of isopropanol–water as the mobile phase. For every compound, parameters were determinated for five concentrations. For chalcones, flavanones, flavones, and 3-hydroxyflavones, concentration ratios of the isopropanol–water mixture were 55%, 60%, 65%, 70%, and 75%, respectively. Concentrations for acetylated derivatives were 50%, 55%, 60%, 65%, and 70%, respectively.

The 44 compounds were dissolved in dichlormethane (1mg/mL) and every solution was applied manually with a capillary four times. Distance between every compound was 1 cm and the migration distance was 8 cm in all cases. Elution of compounds was performed in a developing chamber previously saturated with mobile phase for 30 min at room pressure and temperature. After complete elution, spots were visualized in UV light at wavelengths of 254 nm and 365 nm (see Supplementary Materials). To reduce the errors that may occur, for each concentration three measurementswere made.

Regular retention behavior of investigated compounds was observed. Retention decreases with increasing concentration of organic modifier in the mobile phase. Based on retention factor (R*_f_*), the relative lipophily (R_M_^0^), slope (*b*), chromatographic hydrophobic index (φ_0_), and scores of principal component (PC1/R_M_) were determined.

### 3.2. Computational Evaluation

Schrodinger computational package [39] was used for descriptors computation and the pharmacophore model, while Origin software was used for statistical analysis and representation of data.

In order to computationally assess the lipophilicity and, consecutively, the drug-like properties of the discussed compounds, in silico 3D models were generated starting from 2D chemical formulas. SDF file format was used to store 3D chemical information. Energy minimization, potential energy calculus, and protonation at 310 K at pH 7.4, respectively, were performed using MM2 force field using TINKER software package [40]. Chemical descriptors assessing the lipophilicity were computed using the Schrodinger software.

## 4. Conclusions

From the 44 synthesized compounds, the highest retention capacity was shown by chlorine-substituted *para* hydroxyl-chalcone. Regardless of the basic structure, chlorine substitution of phenylthiazole caused an increase in lipophilicity of the molecule, and the hydroxyl group increased the hydrophilia of the synthesized compounds. Flavanones have a lower retention capacity than the corresponding flavones. Based on the results obtained, it can be stated that lipophilicity is influenced by the basic structure of the molecule and by the nature, position, and number of functional groups on the basic structure. Aromatic groups and H-accepting groups seem to be crucial in explaining lipophilicity of these compounds. All the discussed compounds computationally showed excellent drug-like properties, with good biological membrane absorption factors. Lipophilicity has a crucial role in the biological activity of the concerned compounds and measuring or computing the discussed descriptors represent a key component in the study of drug-like properties of the molecules.

## Supplementary Materials

Supplementary Materials are available online.

## Abbreviations

#stars	number of property or descriptors values that fall outside the 95% range of similar values for known drugs.
%Human	percent human oral absorption
accptHB	estimated number of hydrogen bounds that would be donated,
AlogP	octanol/water partition coefficient
ClQPlogS	conformation independent predicted aquenos solubility
CNS	predicted central nervous system activity
donorHB	estimated number of hydrogen bounds
FISA	a component of SASA
FOSA	hydrophobic component of the SASA
Glob	globularity descriptor
Human	human oral absorbtion
in34	number of atoms in 3 or 4 membered rings
in56	number of atoms in 5 or 6 membered rings
Jm	predicted maximum transdermal transport
metab	number of likely metabolic reactions
noncom	number of ring atoms not able to form conjugated aromatic systems
nonHatm	number of heavy atoms
PEOE1	total positive van der Waals surface area
PISA	number of carbon atoms and the attached hydrogens
Polar	polarity
PSA	polar surface area
QPlogBB	predicted brain blood partition coefficient
QPlogHERG	predicted IC_50_ value for blok HERG chanels
QPlogKhsa	prediction of binding to serum albumin
QplogKp	predicted skin permeability
QPlogPC16	predicted hexadecane gas partition coefficient
QPlogPo/w	predicted octanol water partition coefficient
QPlogPoct	predicted octanolgass partition coefficient
QPlogPw	predicted water gass partition coefficient
Qpolrz	predicted polarizability in cubic angstroms
QPPCaco	predicted Caco-2 cell permeability in nm/s
QPPMDCK	predicted MDCK cell permeability in nm/s
R2	correlation coefficient
Ringatoms	number of atoms in a ring
RM_0_	relative lipophilycity
rtvFG	number of reactive functional groups
Rule	rule of five
SASA	total solvent accessible surface area
S	slope
Volume	total solvent accessible area in cubic Angstroms
WPSA	weakly polar component of the SASA
Φ_0_	chromatographic hydrophobic index
