# Supplementary material for: Lipophilicity as a Central Component of Drug-Like Properties of Chalchones and Flavonoid Derivatives
Source: Molecules. 2019 Apr 17;24(8):1505. doi: 10.3390/molecules24081505 (PMC6515054; doi:10.3390/molecules24081505)
Supplement: Supplementary file 1 [file molecules-24-01505-s001.zip › table of structures.docx]

| **Table 1 Synthesized compounds** | | | | | | | | | | | | | | | | | | | | |
| --- | --- | --- | --- | --- | --- | --- | --- | --- | --- | --- | --- | --- | --- | --- | --- | --- | --- | --- | --- | --- |
| **Thiazole chalcones** | | | | | | | | | | | | | | | | | | | | |
|  | **1a-j** | **R_1_** | | | **R_2_** | | | | **R_3_** | | **1k-t** | | **R_1_** | | | **R_2_** | | | **R_3_** | |
|  | **1a** | **H** | | | **H** | | | | **OH** | | **1k** | | **Cl** | | | **H** | | | **OCH_3_** | |
|  | **1b** | **CH_3_** | | | **H** | | | | **OH** | | **1l** | | **OCH_3_** | | | **H** | | | **OCH_3_** | |
|  | **1c** | **Cl** | | | **H** | | | | **OH** | | **1m** | | **H** | | | **OCH_3_** | | | **OCH_3_** | |
|  | **1d** | **OCH_3_** | | | **H** | | | | **OH** | | **1n** | | **CH_3_** | | | **OCH_3_** | | | **OCH_3_** | |
|  | **1e** | **H** | | | **OH** | | | | **H** | | **1o** | | **Cl** | | | **OCH_3_** | | | **OCH_3_** | |
|  | **1f** | **CH_3_** | | | **OH** | | | | **H** | | **1p** | | **OCH_3_** | | | **OCH_3_** | | | **OCH_3_** | |
|  | **1g** | **Cl** | | | **OH** | | | | **H** | | **1q** | | **H** | | | **OCH_3_** | | | **H** | |
|  | **1h** | **OCH_3_** | | | **OH** | | | | **H** | | **1r** | | **CH_3_** | | | **OCH_3_** | | | **H** | |
|  | **1i** | **H** | | | **H** | | | | **OCH_3_** | | **1s** | | **Cl** | | | **OCH_3_** | | | **H** | |
|  | **1j** | **CH_3_** | | | **H** | | | | **OCH_3_** | | **1t** | | **OCH_3_** | | | **OCH_3_** | | | **H** | |
| **Thiazole flavanones** | | | | | | | | | | | | | | | | | | | | |
|  | **2a-d** | | | | | | | | | | **R_1_** | | | | | | | | | |
|  | **2a** | | | | | | | | | | **H** | | | | | | | | | |
|  | **2b** | | | | | | | | | | **CH_3_** | | | | | | | | | |
|  | **2c** | | | | | | | | | | **Cl** | | | | | | | | | |
|  | **2d** | | | | | | | | | | **OCH_3_** | | | | | | | | | |
| **Thiazole flavones and 3-hydroxyflavones** | | | | | | | | | | | | | | | | | | | | |
|  | **3a-d** | | | **R_1_** | | | **R_2_** | | | | **3e-h** | | | | **R_1_** | | | **R_2_** | | |
|  | **3a** | | | **H** | | | **H** | | | | **3e** | | | | **H** | | | **OH** | | |
|  | **3b** | | | **CH_3_** | | | **H** | | | | **3f** | | | | **CH_3_** | | | **OH** | | |
|  | **3c** | | | **Cl** | | | **H** | | | | **3g** | | | | **Cl** | | | **OH** | | |
|  | **3d** | | | **OCH_3_** | | | **H** | | | | **3h** | | | | **OCH_3_** | | | **OH** | | |
| **Acetylated derivatives of chalcones** | | | | | | | | | | | | | | | | | | | | |
|  | **4a-d** | | **R_1_** | | | **R_2_** | | | | **R_3_** | **4e-h** | **R_1_** | | | | | **R_2_** | | | **R_3_** |
|  | **4a** | | **H** | | | **H** | | | | **OAc** | **4e** | **H** | | | | | **OAc** | | | **H** |
|  | **4b** | | **CH3** | | | **H** | | | | **OAc** | **4f** | **CH3** | | | | | **OAc** | | | **H** |
|  | **4c** | | **Cl** | | | **H** | | | | **OAc** | **4g** | **Cl** | | | | | **OAc** | | | **H** |
|  | **4d** | | **OCH3** | | | **H** | | | | **OAc** | **4h** | **OCH3** | | | | | **OAc** | | | **H** |
| **Acetylated derivatives of flavones** | | | | | | | | | | | | | | | | | | | | |
|  | **4i-j** | **R_1_** | | | | | | **R_2_** | | | **4k-l** | | | **R_1_** | | | **R_2_** | | | |
|  | **4i** | **H** | | | | | | **OAc** | | | **4k** | | | **Cl** | | | **OAc** | | | |
|  | **4j** | **CH3** | | | | | | **OAc** | | | **4l** | | | **OCH_3_** | | | **OAc** | | | |
